# Influence of Pd Doping on Electrical and Thermal Properties of *n*-Type Cu_0.008_Bi_2_Te_2.7_Se_0.3_ Alloys

**DOI:** 10.3390/ma12244080

**Published:** 2019-12-06

**Authors:** Se Yun Kim, Hyun-Sik Kim, Kyu Hyoung Lee, Hyun-jun Cho, Sung-sil Choo, Seok-won Hong, Yeseong Oh, Yerim Yang, Kimoon Lee, Jae-Hong Lim, Soon-Mok Choi, Hee Jung Park, Weon Ho Shin, Sang-il Kim

**Affiliations:** 1 Samsung Electronics, Suwon 16678, Korea; ksyvip@gmail.com; 2 Department of Materials Science and Engineering, Hongik University, Seoul 04066, Korea; hyunsik.kim@hongik.ac.kr; 3 Department of Materials Science and Engineering, Yonsei University, Seoul 03722, Korea; khlee2018@yonsei.ac.kr; 4 Department of Materials Science and Engineering, University of Seoul, Seoul 02504, Korea; joshua4150@uos.ac.kr (H.-j.C.); cntjdtlf111@uos.ac.kr (S.-s.C.); seokown4926@uos.ac.kr (S.-w.H.); asdf85205@uos.ac.kr (Y.O.); yyl0201@uos.ac.kr (Y.Y.); 5 Department of Physics, Kunsan National University, Gunsan 54150, Korea; kimoon.lee@kunsan.ac.kr; 6 Department of Materials Science and Engineering, Gachon University, Seongnam 13120, Korea; limjh@gachon.ac.kr; 7 School of Energy, Materials and Chemical Engineering, Korea University of Technology and Education, Cheonan 31253, Korea; smchoi@koreatech.ac.kr; 8 Department of Materials Science and Engineering, Dankook University, Cheonan 31116, Korea; parkjang@dankook.ac.kr; 9 Department of Electronic Materials Engineering, Kwangwoon University, Seoul 01897, Korea

**Keywords:** thermoelectric, Pd doping, effective mass, bipolar thermal conductivity, phonon scattering

## Abstract

Doping is known as an effective way to modify both electrical and thermal transport properties of thermoelectric alloys to enhance their energy conversion efficiency. In this project, we report the effect of Pd doping on the electrical and thermal properties of *n*-type Cu_0.008_Bi_2_Te_2.7_Se_0.3_ alloys. Pd doping was found to increase the electrical conductivity along with the electron carrier concentration. As a result, the effective mass and power factors also increased upon the Pd doping. While the bipolar thermal conductivity was reduced with the Pd doping due to the increased carrier concentration, the contribution of Pd to point defect phonon scattering on the lattice thermal conductivity was found to be very small. Consequently, Pd doping resulted in an enhanced thermoelectric figure of merit, *zT*, at a high temperature, due to the enhanced power factor and the reduced bipolar thermal conductivity.

## 1. Introduction

Thermoelectric alloys have attracted attention in recent decades because these materials can convert a temperature gradient directly into electrical energy. Bismuth telluride (Bi_2_Te_3_)-based alloys are currently the most used bulk thermoelectric alloys near room temperature [1,2]. However, the broader use of Bi_2_Te_3_-based alloys is still limited by the rather low thermoelectric conversion performance, evaluated as the thermoelectric figure of merit *zT* = *σ*·*S*^2^·*T*/*κ_tot_*, where *σ*, *S*, *T,* and *κ_tot_* are the electrical conductivity, Seebeck coefficient, temperature, and total thermal conductivity, respectively. In fact, the *zT* of *n*-type Bi_2_(Te,Se)_3_ alloys remains below 1, while values significantly higher than 1 have often been reported for *p*-type (Bi,Sb)_2_Te_3_ alloys.

Doping is an effective approach to improving the *zT* of Bi_2_Te_3_ alloys by adjusting the electrical transport properties or reducing the *κ* through the introduction of additional point defects [3,4,5,6,7,8,9,10,11]. The *zT* of *p*-type (Bi,Sb)_2_Te_3_ alloys can easily be enhanced using substitutional dopants [3,4,5]. Meanwhile, the influence of doping on *n*-type Bi_2_(Te,Se)_3_ alloys has not been investigated as much as that of doping on *p*-type (Bi,Sb)_2_Te_3_ alloys. It has been found that Cu intercalation in *n*-type Bi_2_(Te,Se)_3_ alloys is a very effective approach to reducing the lattice thermal conductivity (*κ_latt_*) by introducing additional point defect scattering centers [12]. However, the accompanying modification of carrier transport properties with the *κ_latt_* reduction may reduce the power factor, resulting in *zT* reduction.

Co-doping of two different substituents was also suggested to further decrease the *κ_latt_* while enhancing the power factors in some other thermoelectric materials, such as SnTe and PbTe [13,14,15,16]. In *p*-type (Bi,Sb)_2_Te_3_ alloys, it was found that the co-doping of Ag and Ga reduced the *κ_latt_* further compared to that of single doped materials [17], while the power factor can be maintained.

Herein, we investigated the effect of additional Pd substitutional doping on the electrical transport properties and thermal conductivities of Cu-doped *n*-type Bi_2_(Te,Se)_3_, Cu_0.008_Bi_2_Te_2.7_Se_0.3_. The Pd was anticipated to scatter phonons effectively due to the large mass and ionic radius differences between Pd and Bi (M_Pd_ = 106.42 u, M_Bi_ = 208.98 u, *r_Pd_* = 90 pm, *r_Bi_* = 117 pm). Pd doping increased the electron concentration, electrical conductivity, and power factors. However, the contribution of Pd to additional point defect scattering centers on the lattice thermal conductivity was found to be rather small. As a result, *zT* enhancement due to Pd doping was observed at high temperatures. To investigate the reason for the limited effectiveness of substitutional Pd doping in reducing the *κ_latt_* value in *n*-type Bi_2_(Te,Se)_3_ alloys, the electronic transport properties were analyzed using a single parabolic band model [18], and the reduction in *κ_latt_* was quantitatively predicted using the Debye–Callaway model [19].

## 2. Materials and Methods

The Cu_0.008_Bi_2_Te_2.7_Se_0.3_ reference sample and a series of Pd-doped Cu_0.008_Pd*_x_*Bi_2-*x*_Te_2.7_Se_0.3_ (*x* = 0.002, 0.004, 0.01, and 0.02) samples were synthesized by a conventional solid state reaction for 10 h at 1423 K, using high-purity (99.999%) raw materials. The synthesized ingots were ball-milled using a 8000M Mixer/Mill high-energy ball mill (SPEX SamplePrep, Metuchen, NJ, USA) for 10 min, and sieved powders under 45 µm were consolidated by spark plasma sintering at 723 K and 50 MPa for 2 min. Then, the temperature-dependent *S* and *σ* parameters were measured over the temperature range between room temperature and 480 K in a direction perpendicular to the pressing direction (ZEM-3, Advanced-RIKO, Yokohama, Japan). The carrier concentrations were determined by Hall measurements in van der Pauw configuration, in a magnetic field of 0.5 T (AHT-55T5, Ecopia, Anyang, South Korea) in the same direction. The *κ* values of the samples were calculated from their theoretical density (*ρ*_s_), heat capacity (*C*_p_), and thermal diffusivity (*λ*) values (*κ* = *ρ*_s_⋅*C*_p_⋅*λ*), measured along the same direction.

## 3. Results and Discussion

**Figure 1:**
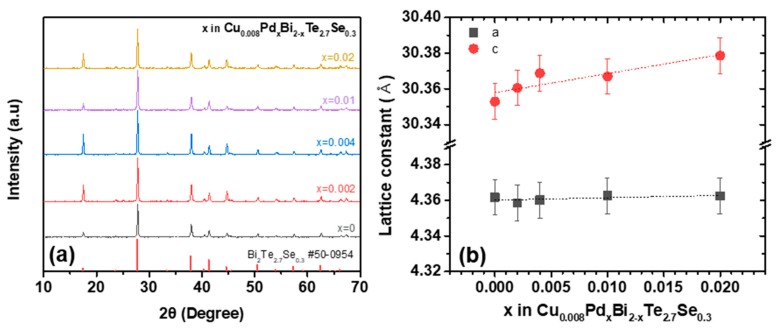
(**a**) X-ray diffraction patterns and (**b**) calculated lattice parameters *a* and *c* of Cu_0.008_Pd*_x_*Bi_2-*x*_Te_2.7_Se_0.3_ (*x* = 0, 0.002, 0.004, 0.01, and 0.02).

Figure 1a shows the X-ray Diffraction (XRD) patterns of the investigated series of Cu_0.008_Pd*_x_*Bi_2-*x*_Te_2.7_Se_0.3_. All samples showed single phases without impurities. The lattice parameters *a* and *c* are shown in Figure 1b, which reveals that the *c* parameter generally increased with the Pd doping, while *a* remained largely unchanged upon doping. The systematic change in the *c* parameter implies that substitutional doping was successfully achieved.

**Figure 2:**
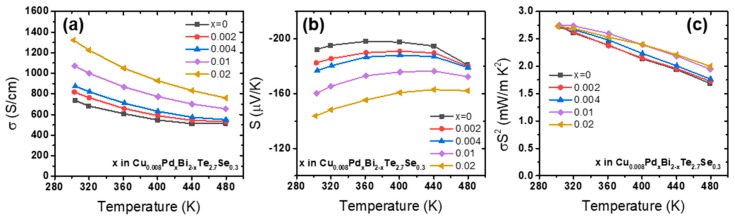
(**a**) *σ*, (**b**) *S*, and (**c**) power factor of Cu_0.008_Pd*_x_*Bi_2-*x*_Te_2.7_Se_0.3_ (*x* = 0, 0.002, 0.004, 0.01, and 0.02).

The measured *σ* and *S* values of the Pd-doped Cu_0.008_Pd*_x_*Bi_2-*x*_Te_2.7_Se_0.3_ (*x* = 0, 0.002, 0.004, 0.01, and 0.02) are shown in Figure 2a,b. The *σ* value of the undoped sample was about 740 S/cm at 300 K, and substantially increased to 1320 S/cm for *x* = 0.02. On the other hand, the magnitude of the *S* values at 300 K decreased from −192 to −144 µV/K. As a result, the power factor (*S*^2^·*σ*) at 300 K remained unchanged (around 2.73 mW/m·K^2^) regardless of the Pd doping level (Figure 2c). However, an enhancement in the power factor was observed at high temperatures upon Pd doping. For example, at 480 K the power factor was enhanced by 19%, from 1.68 to 2.00 mW/m·K^2^.

**Figure 3:**
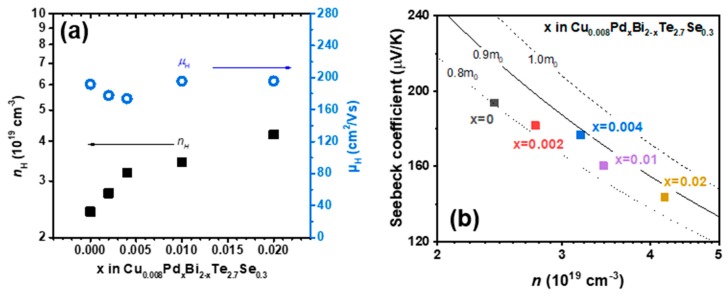
(**a**) Measured carrier concentrations and mobilities and (**b**) Pisarenko plot.

Figure 3a shows the electron carrier concentration (*n*_H_) and mobility (*µ*_H_) measured for the Cu_0.008_Pd*_x_*Bi_2-*x*_Te_2.7_Se_0.3_ samples at 300 K. The *n*_H_ gradually increased with the Pd doping, with *n*_H_ values of 2.4, 2.8, 3.2, 3.4, and 4.2 × 10^19^ cm^−3^ for *x* = 0, 0.002, 0.004, 0.01, and 0.02, respectively. On the other hand, the *µ*_H_ values did not change significantly. Therefore, the increase in *σ* upon Pd doping is mainly due to the increased *n*_H_ values. Figure 3b shows the Pisarenko plot of the samples, displaying the *S* of samples as a function of *n*_H_ at 300 K. The solid lines were obtained for different effective masses (m^*^ = 0.8, 0.9, and 1.0 *m*_0_, where *m*_0_ is the electron mass) using Equation (1):

(1) $$S=\frac{8\pi^{2}k_{B}{}^{2}}{3eh^{2}}{\left(\frac{\pi }{3n}\right)}^{2/3}m^{*}T$$

where *e*, *h*, and *k*_B_ are the elementary charge, Planck’s constant, and Boltzmann constant, respectively. The m^*^ values of all samples, deduced using Equation (1), are plotted in Figure 3b. As shown in the figure, Pd doping resulted in slightly increased m^*^ values, indicating that the electronic structure of the conduction band of Cu_0.008_Bi_2_Te_2.7_Se_0.3_ was slightly modified favorably for *S*.

**Figure 4:**
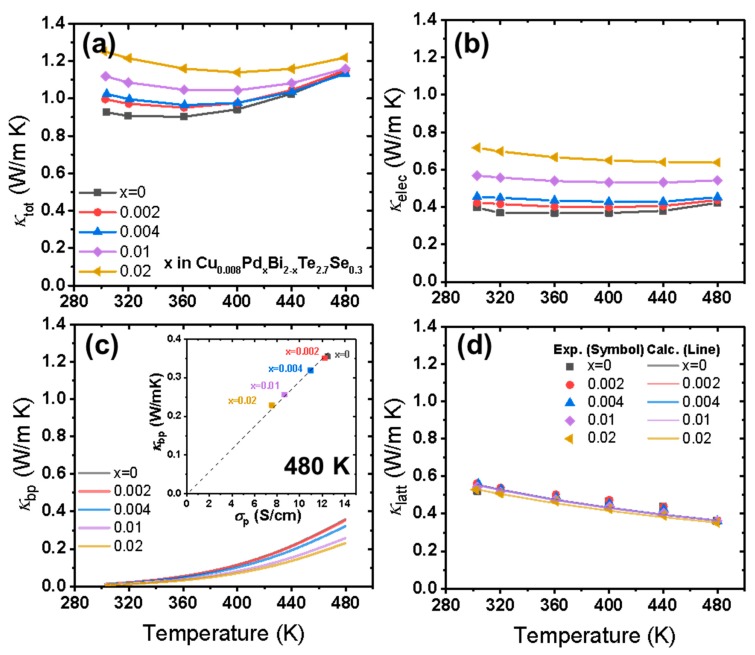
(**a**) *κ_tot_* (*κ_tot_* = *κ_elec_* + *κ_latt_* + *κ_bp_*), (**b**) *κ_elec_*, (**c**) *κ_bp_*, and (**d**) *κ_latt_*. The inset of (**c**) shows the linear relationship between *κ_bp_* and *σ*_p_.

Figure 4a shows the measured *κ_tot_* of the Pd-doped Cu_0.008_Bi_2_Te_2.7_Se_0.3_ samples, revealing that the *κ_tot_* values gradually increased with the doping level. In order to understand these changes, we analyzed the contributions to *κ_tot_*, given by the following equation:

(2) $$\kappa_{tot}=\kappa_{elec}+\kappa_{bp}+\kappa_{latt}$$

where *κ_elec_* and *κ_bp_* are the electronic and bipolar thermal conductivities, respectively. First, *κ_elec_* was calculated using the equation for the Lorenz number (*L*, expressed as a simple function with *S* in Equation (3)) [20], and the results are shown in Figure 4b.

(3) $$L=1.5+\exp\left(-\frac{\left|S\right|}{116}\right)$$

Equation (3) describes the relationship between the *L* and *S* in a simple function, based on a single parabolic band model [20]. The *κ_elec_* values increased as the electrical conductivity increased with Pd doping, straightforwardly with the increased carrier concentration (Figure 3a). At 300 K, *κ_elec_* showed a significant increase from 0.4 to 0.7 W/m·K.

The *κ_bp_* parameter, related to the bipolar electronic transport properties, can be estimated based on a single parabolic band model and the Boltzmann transport equation (Equation (4)):

(4) $$\kappa_{bp}=\left(S_{p}{}^{2}\sigma_{p}+S_{n}{}^{2}\sigma_{n}-S^{2}\sigma \right)\;T$$

where *σ_p_* and *σ_n_* are the electrical conductivities of the valence (*p*) and conduction (*n*) bands (VB and CB, respectively), while *S_p_* and *S_n_* are the Seebeck coefficients for the VB and CB, respectively.

The details of the *κ_bp_* calculations are provided with the two-band model analysis in the Supplementary Materials, while the results of the calculations are shown in Figure 4c. The *κ_bp_* value was gradually reduced from 0.36 to 0.23 W/m·K at 480 K, which represents a 36% decrease. The decrease in *κ_bp_* is also mostly related to the increased concentration of electron carriers, which are the majority carriers. Therefore, the influence of the minority carriers is reduced. The inset of Figure 4c highlights a linear relationship between the *κ_bp_* and *σ_p_* values at 480 K [21]. The *σ_p_* values estimated from the two-band model are provided in the Supplementary Materials and Table S1.

Then, the *κ_latt_* were deduced by subtracting the *κ_elec_* and *κ_bp_* values from the measured *κ_tot_*, and are shown as symbols in Figure 4d. The *κ_latt_* (symbols in Figure 4d) was fitted to the theoretical *κ_latt_* (lines in Figure 4d) using the Debye-Callaway equation:

(5) $$\kappa_{latt}=\;\frac{k_{B}}{2\pi^{2}\nu }{\left(\frac{k_{B}T}{\hbar }\right)}^{3}\int_{0}^{\theta_{D}/T}\tau_{tot}\left(z\right)\frac{z^{4}e^{z}}{{\left(e^{z}-1\right)}^{2}}dz$$

where *τ_tot_*, *θ_D_*, *v*, and *ħ* are the total phonon relaxation time, Debye temperature, phonon group velocity, and Planck constant divided by 2*π*, respectively, while *z = ħω*/*k_B_T* (*ω* = phonon frequency). Therefore, the determined *τ_tot_*(*z*) values describe the theoretical *κ_latt_*, whereas *τ_tot_*(*z*) can be estimated from the individual phonon relaxation times (*τ_i_*) for scattering mechanisms, based on Matthiessen’s equation (Equation (6)):

(6) $$\tau_{\mathrm{total}}{\left(z\right)}^{-1}=\underset{i}{\sum }\tau_{i}{\left(z\right)}^{-1}=\;\tau_{U}{\left(z\right)}^{-1}+\;\tau_{B}{\left(z\right)}^{-1}+\;\tau_{PD}{\left(z\right)}^{-1}.$$

For scattering by point defects, which is the dominant mechanism in the present Pd doping case, the phonon relaxation time can be described using the scattering parameter (Г) within *τ_PD_*, as shown in Equations (7) and (8):

(7) $$\tau_{PD}{}^{-1}=P\;f\left(1-f\right)\;\omega^{4}=\;\frac{V\omega^{4}}{4\pi v^{3}}Г$$

(8) $$Г=f\left(1-f\right)\left[{\left(\frac{\Delta M}{M}\right)}^{2}+\frac{2}{9}{\left\{\left(G+6.4\gamma \right)\frac{1+r}{1-r}\right\}}^{2}{\left(\frac{\Delta a}{a}\right)}^{2}\right].$$

In Equation (7), *P* and *f* are a fitting parameter and substituting fraction, respectively. In Equation (8), Δ*M* and Δ*a* are the difference in mass and lattice constant between the two constituents of an alloy. The *G* and γ represent the ratio of the fractional change in the bulk modulus to the local bond length and the Grüneisen parameter, while *r* is the Poisson ratio. Further details of the calculation were not included here, because we found no differences in the *κ_latt_* values.

The theoretical *κ_latt_* is shown as solid lines in Figure 4d, along with the experimental *κ_latt_* (symbols). The experimental or theoretical *κ_latt_* values show rather small changes with the doping level, despite reaching a maximum at *x* = 0.02, implying that only minor additional scattering originated from the doped Pd. This is a peculiar result, as there is much evidence of additional point defect scattering upon substitutional doping. Due to the effect of the mass and lattice constant differences between two constituents of an alloy, described by Equation (8), we would expect a rather large additional contribution from phonon scattering, due to the large mass and ionic radius differences between Pd and Bi (*M_Pd_* = 106.42 u, *M_Bi_* = 208.98 u, *r_Pd_* = 90 pm, *r_Bi_* =117 pm). Despite the rather large Δ*M* and Δ*a* values, we did not observe significant additional scattering due to the Pd doping. A possible explanation would be that intercalated Cu and Te/Se disorder already provide enough point defect scattering, so that Pd substitution would not contribute further in reducing *κ_latt_*. Scattering from Cu is known to be rather effective [12]. Consequently, the *κ_tot_* value at 300 K showed a significant increase due to the increased *κ_elec_*, whereas that at 480 K increased only slightly, together with the decrease in *κ_bp_*, seen in Figure 4a.

**Figure 5:**
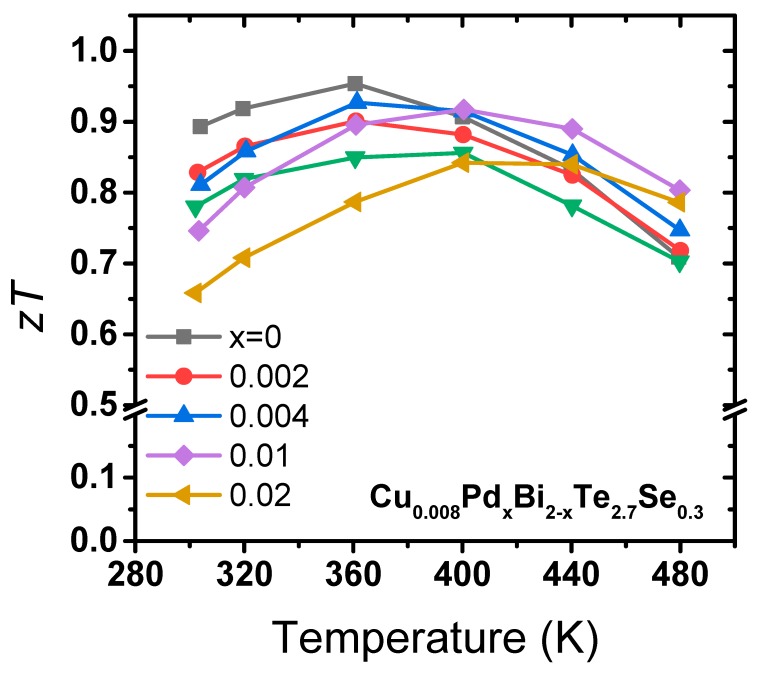
*zT* values of Cu_0.008_Pd*_x_*Bi_2-*x*_Te_2.7_Se_0.3_ (*x* = 0, 0.002, 0.004, 0.01, and 0.02).

Figure 5 shows the *zT* values of all samples. At low temperatures, the *zT* values were reduced, mainly due to the *κ_tot_* increase. However, at higher temperatures (over 400 K), enhanced *zT* values were observed for intermediate Pd doping levels of *x* = 0.004 and 0.01. This is due to the enhanced power factors, along with the fact that *κ_tot_* did not increase significantly despite the *κ_elec_* increase. For instance, the *zT* at 480 K increased from 0.70 to 0.79 in the *x* = 0.01 case. However, no clear Pd doping-induced enhancement in *zT* was observed at doping levels higher than *x* = 0.01, due to the simultaneous increase in *κ_elec_* and *κ_tot_*, resulting from an excessive increase in electron carrier concentration. We found that moderate doping of Pd with levels of x = 0.004 to 0.01 in *n*-type Cu_0.008_Bi_2_Te_2.7_Se_0.3_ can be effective in enhancing the power factor. However, the Pd doping in Cu-doped *n*-type Bi_2_(Te,Se)_3_ did not further reduce *κ*_latt_ despite the rather large Δ*M* and Δ*a* values.

## 4. Conclusions

We studied the influence of Pd substitution in *n*-type Cu-doped Bi_2_Te_2.7_Se_0.3_, Cu_0.008_Bi_2_Te_2.7_Se_0.3_, by analyzing the electrical and thermal properties of a series of *n*-type Cu_0.008_Pd*_x_*Bi_2-*x*_Te_2.7_Se_0.3_ alloys (*x* = 0, 0.002, 0.004, 0.01, and 0.02) based on a single parabolic band and Debye-Callaway models. As the Pd doping increased, the electron carrier concentration and electrical conductivity increased simultaneously. The power factor was also enhanced, especially at higher temperatures. The bipolar conduction in the Pd-doped Cu_0.008_Bi_2_Te_2.7_Se_0.3_ samples was reduced; in particular, the bipolar thermal conductivity showed a significant decrease from 0.36 W/m·K in the undoped sample to 0.24 W/m·K in the *x* = 0.02 doped sample at 480 K. However, the analysis of the lattice thermal conductivity showed that substitutional Pd is not very effective in enhancing phonon scattering when interstitial Cu and Se/Te disorder are already present. Consequently, enhanced *zT* values at temperatures higher than 400 K were observed for the *x* = 0.004 and 0.01 doped samples.

## Supplementary Materials

The following are available online at https://www.mdpi.com/1996-1944/12/24/4080/s1, Table S1 Band parameters of Pd-doped Cu_0.008_Pd*_x_*Bi_2-*x*_Te_2.7_Se_0.3_ samples (*x* = 0, 0.002, 0.004, 0.01, and 0.02) calculated using the two-band model.
