# Supplementary material for: Influence of Pd Doping on Electrical and Thermal Properties of n-Type Cu0.008Bi2Te2.7Se0.3 Alloys
Source: Materials (Basel). 2019 Dec 6;12(24):4080. doi: 10.3390/ma12244080 (PMC6947468; doi:10.3390/ma12244080)
Supplement: Supplementary file 1 [file materials-12-04080-s001.pdf]

materials

MDPI

Supplementary Materials

Influence of Pd Doping on Electrical and Thermal Properties of *n*-Type $Cu_{0.008}Bi_2Te_{2.7}Se_{0.3}$ Alloys

Se Yun Kim, Hyun-Sik Kim, Kyu Hyoung Lee, Hyun-jun Cho, Sung-sil Choo, Seok-won Hong, Yeseong Oh, Yerim Yang, Kimoon Lee, Jae-Hong Lim, Soon-Mok Choi, Hee Jung Park, Weon Ho Shin*, and Sang-il Kim*

S1. Theoretical analysis of electronic transport properties of $Cu_{0.008}Pd_xBi_{2-x}Te_{2.7}Se_{0.3}$ (x = 0, 0.002, 0.004, 0.01, and 0.02)

Quantitative analysis based on the two-band model can provide the transport parameters for both majority and minority carriers (corresponding to electrons and holes, respectively, in the present case). To estimate the separate contributions of majority and minority carriers, the individual σ_i and S_i for the valence (VB) and conduction (CB) bands (where $i = p$ or n for the VB or CB, respectively) were estimated from the Boltzmann transport equations (Equations (S1) and (S2)):

$$\sigma = \sigma_\mathrm{p} + \sigma_\mathrm{n} \quad \text{(S1)}$$

$$S = \frac{\sigma_\mathrm{p} S_\mathrm{p} - \sigma_\mathrm{n}|S_\mathrm{n}|}{\sigma_\mathrm{p} + \sigma_\mathrm{n}} \quad \text{(S2)}$$

where σ_p and σ_n are the electrical conductivities of the VB (p) and CB (n), while S_p and S_n are the Seebeck coefficients for the VB and CB, respectively. Using the two-band model, the deformation potential (E_{def}) and effective mass (m^*) values for the VB and CB were fitted to the total S and σ at 300 K (Figures 3(a) and 3(b)). The E_{def} parameter describes the carrier–phonon interaction, i.e., a band with a large E_def has low mobility. For the calculation, we used a band gap (E_g) value of 0.2 eV ($Bi_2Te_{2.7}Se_{0.3}$ alloy [S1]) and a longitudinal elastic modulus (C_l) of 64.6 GPa. The carrier concentrations for the VB and CB were also calculated based on Equation (S3):

$$R_{H_{tot}} = \frac{R_{H_p}{\sigma_p}^2 + R_{H_n}{\sigma_n}^2}{\left(\sigma_p + \sigma_n\right)^2} \quad \text{(S3)}$$

where R_{Htot}, R_{Hp}, and R_{Hn} are the Hall coefficients for total conduction, VB, and CB, respectively; the R_{Hp} and R_{Hn} parameters were converted to electron and hole concentrations. All calculated parameters and changes in the E_def and m^*values of VB and CB with Pd doping are shown in Table S1.

Table S1. Band parameters of Pd-doped $Cu_{0.008}Pd_xBi_{2-x}Te_{2.7}Se_{0.3}$ samples (x = 0, 0.002, 0.004, 0.01, and 0.02) calculated using the two-band model.

Band parameters	x = 0	x = 0.002	x = 0.004	x = 0.01	x = 0.02
Conduction band (CB) E_def (eV)	7.65	7.82	7.89	7.92	8
CB m^* (in m_0)	1.03	1.04	1.06	0.99	0.96
$R_{\mathrm{H},n}$ (cm^3/C)	−0.2986	−0.2610	−0.2236	−0.2064	−0.1672
Electron concentration (cm^{-3})	2.41 × 10^{19}	2.75 × 10^{19}	3.19 × 10^{19}	3.43 × 10^{19}	4.19 × 10^{19}
σ_n @ 300K (S/cm)	779.46	815.86	871.14	1070.56	1319.38
σ_n @ 480K (S/cm)	431.81	452.90	483.99	599.73	748.50
Valence band (VB) E_def (eV)	20.9	19.8	19.5	19.8	18.2
VB m^* (in m_0)	1	1	1	1	1
$R_{\mathrm{H},p}$ (cm^3/C)	784.20	914.74	1079.91	1386.60	2015.44
Hole concentration (cm^{-3})	0.96 × 10^{16}	0.83 × 10^{16}	0.70 × 10^{16}	0.54 × 10^{16}	0.37 × 10^{16}

σ_p @ 300K (S/cm)	0.3297	0.3149	0.2750	0.2077	0.1691
σ_p @ 480K (S/cm)	12.4581	12.2007	10.9894	8.6501	7.5491

E_{def} = deformation potential, m^* = density-of-states effective mass (m_0 = electron mass).

References

S1. Lee, K.H.; Kim, S.I.; Mun, H.; Ryu, B.K.; Choi, S.M.; Park, H.J.; Hwang, S.W.; Kim, S.W. Enhanced thermoelectric performance of n-type $Cu_{0.008}Bi_2Te_{2.7}Se_{0.3}$ by band engineering. *J. Mater. Chem. C*. **2015**, *3*, 10604; DOI:10.1039/c5tc01731a

© 2019 by the authors. Submitted for possible open access publication under the terms and conditions of the Creative Commons Attribution (CC BY) license (http://creativecommons.org/licenses/by/4.0/).
